# Supplementary material for: Roles of Lipopolysaccharide Glycosyltransferases in Maintenance of Helicobacter pylori Morphology, Cell Wall Permeability, and Antimicrobial Susceptibilities
Source: Int J Mol Sci. 2023 Jul 12;24(14):11381. doi: 10.3390/ijms241411381 (PMC10379358; doi:10.3390/ijms241411381)
Supplement: Supplementary file 1 [file ijms-24-11381-s001.zip › ijms-2444800-supplementary.pdf]

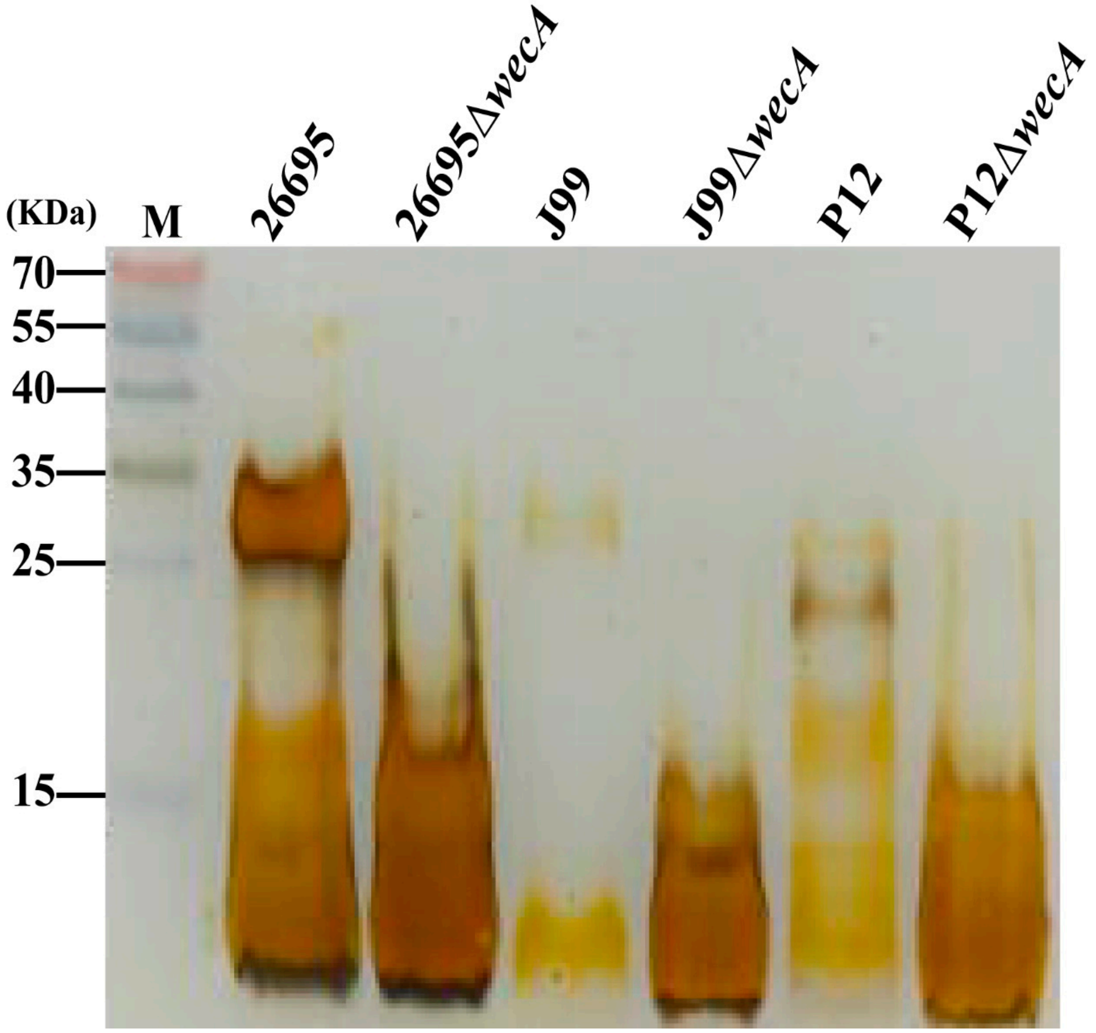

Figure S1. LPS structural characterization of *H. pylori* wild-type and Δ*wecA* mutants. LPSs of 26695, J99, P12 wild-type and their Δ*wecA* mutants obtained by the deletion of O-antigen initial biosynthesis *wecA* gene were extracted and detected by silver staining. The O-antigen of all the Δ*wecA* mutants were lost compared with that of the wild-type strains. M, molecular weight marker.
